# Inhibition of colorectal cancer genomic copy number alterations and chromosomal fragile site tumor suppressor FHIT and WWOX deletions by DNA mismatch repair

**DOI:** 10.18632/oncotarget.17776

**Published:** 2017-05-10

**Authors:** Sohail Jahid, Jian Sun, Ozkan Gelincik, Pedro Blecua, Winfried Edelmann, Raju Kucherlapati, Kathy Zhou, Maria Jasin, Zeynep H. Gümüş, Steven M. Lipkin

**Affiliations:** ^1^ Departments of Medicine and Genetic Medicine, Weill Cornell Medicine, 10021, NY, USA; ^2^ Department of Cell Biology and Department of Genetics, Albert Einstein College of Medicine of Yeshiva University, 10461, NY, USA; ^3^ Department of Genetics, Harvard Medical School, 02115, Boston, MA, USA; ^4^ Department of Biostatistics and Epidemiology, Weill Cornell Medical College, 10021, NY, USA; ^5^ Developmental Biology Program, Memorial Sloan Kettering Cancer Center, 10065, NY, USA; ^6^ Department of Genetics and Genomic Sciences, Icahn School of Medicine at Mount Sinai, 10029, NY, USA; ^7^ Icahn Institute for Genomics and Multiscale Biology, Icahn School of Medicine at Mount Sinai, 10029, NY, USA; ^8^ Division of Clinical Genetics, Memorial Sloan Kettering Cancer Center, 10065, NY, USA

**Keywords:** mismatch repair, homologous recombination, homeologous recombination, colorectal cancer

## Abstract

Homologous recombination (HR) enables precise DNA repair after DNA double strand breaks (DSBs) using identical sequence templates, whereas homeologous recombination (HeR) uses only partially homologous sequences. Homeologous recombination introduces mutations through gene conversion and genomic deletions through single-strand annealing (SSA). DNA mismatch repair (MMR) inhibits HeR, but the roles of mammalian MMR MutL homologues (MLH1, PMS2 and MLH3) proteins in HeR suppression are poorly characterized. Here, we demonstrate that mouse embryonic fibroblasts (MEFs) carrying *Mlh1*, *Pms2*, and *Mlh3* mutations have higher HeR rates, by using 7,863 uniquely mapping paired direct repeat sequences (DRs) in the mouse genome as endogenous gene conversion and SSA reporters. Additionally, when DSBs are induced by gamma-radiation, *Mlh1*, *Pms2* and *Mlh3* mutant MEFs have higher DR copy number alterations (CNAs), including DR CNA hotspots previously identified in mouse MMR-deficient colorectal cancer (dMMR CRC). Analysis of The Cancer Genome Atlas CRC data revealed that dMMR CRCs have higher genome-wide DR HeR rates than MMR proficient CRCs, and that dMMR CRCs have deletion hotspots in tumor suppressors FHIT/WWOX at chromosomal fragile sites *FRA3B* and *FRA16D* (which have elevated DSB rates) flanked by paired homologous DRs and inverted repeats (IR). Overall, these data provide novel insights into the MMR-dependent HeR inhibition mechanism and its role in tumor suppression.

## INTRODUCTION

DNA mismatch repair (MMR) is pivotal in maintaining genomic stability in both prokaryotes and eukaryotes. There are nine mammalian MMR genes, including E. coli MutS homologues (*MSH6*), MutL homologues (*MLH1, MLH3*) and yeast post-meiotic segregation homologues (*PMS*1 and *PMS2*) [1–5]. In humans, *MLH1*, *MSH2*, *MSH6* and *PMS2* mutations increase susceptibility to multiple malignancies, most notably colorectal and endometrial cancers [1]. Mechanistically, mammalian MSH2-MSH6 and MSH2-MSH3 complexes form ‘sliding clamps’ that scan genomic DNA for sequence mismatches [6–9]. MSH2-MSH6 complex binds to single base-base mismatches and small insertions/deletion loops (IDLs), whereas MSH2-MSH3 complex is involved in the repair of larger IDLs [1] (IDL repair deficiency is commonly referred to as Microsatellite Instability, MSI). The MSH proteins interact with multiple proteins including the mammalian *E.coli* MutL homologues (*MLH*) and yeast post-meiotic segregation (*PMS*) homologue proteins, which have significant amino acid identity and structural similarity to the MLH proteins, among others (reviewed in [1]).

The MLH1-PMS2 complex is the primary MutL heterodimer that interacts with both MSH2-MSH6 and MSH2-MSH3 complexes, and is the only heterodimer that participates in repair of single base substitutions. There is partial redundancy in the functions of MLH1-PMS2 and MLH1-MLH3 complexes in IDL repair and DNA damage response [10, 11]. In meiosis, both MLH1-PMS2 and MLH1-MLH3 complexes promote recombination-mediated cross-over events [12–14], and MLH1-PMS2 also plays roles in antibody class switch recombination [15]. A third MutL complex, MLH1-PMS1, has also been reported, but its role in mammalian MMR is yet to be clearly defined.

HR is a critical pathway for repair of DNA double-strand breaks (DSBs). Briefly, in mammals recA-like protein (Rad51) forms complexes with single-stranded DNA (ssDNA) strands. Rad51-ssDNA filaments interact with double stranded DNA (dsDNA) and pair when homologous sequence contacts are made. Subsequently, strand exchange occurs and a hybrid dsDNA/ssDNA complex called a D-loop is formed that is processed by multiple HR sub-pathways, including double-strand break repair (DSBR), synthesis dependent strand annealing (SDSA) and break-induced replication (BIR) [16]. DSBR, SDSA and BIR all produce copy number-neutral HR repair. In contrast, when repetitive sequences flank a DSB, the mechanism of single strand annealing (SSA) can also be used for DSB repair [17]. SSA involves direct annealing between misaligned homologous repeat sequences and ssDNA recession. Consequently, SSA at repetitive homologous motifs causes deletion of one repeat sequence and the intervening sequences between repeats, resulting in deletion copy number alterations (CNAs). When flanked by direct repeats (DRs) or inverted repeats, DSBs stimulate HR and SSA several hundred fold [18].

Relatively small degrees of sequence heterogeneity can alter the frequency of DSB-induced HR and SSA in mammalian cells. For example, HR is decreased by >80% for DRs that are 1.2% divergent [19]. When the donor and recipient sequences are highly similar but not identical (homeologous), the repair process is referred as homeologous recombination (HeR) [16]. MMR proteins play important roles in preventing HeR and SSA. Specifically, MMR recognizes and repairs mismatches in DNA heteroduplex regions (regions that are formed by annealing single strands from different sources), removes non-homologous tails during HeR repair and disrupts HeR repair via heteroduplex rejection or anti-recombination (recently reviewed in [16]).

MMR suppression of HeR was first observed in bacteria [20, 21] where it prevents rotation of mismatched DNA strands [22], and was subsequently found to occur in eukaryotes [23]. In the yeast *Saccharomyces cerevisiae*, functional mutations of *Msh2p, Msh6p, Mlh1p or Pms1p* (orthologues of mammalian *PMS2*) increase HeR between integrated reporters containing DRs with mismatch heterology [23–26], yet roles for Mlh2p/Mlh3p are poorly characterized. Additionally, individual functional mutations of *Msh2p, Mlh1p* or compound mutations of the three *Mlh1p* heterodimeric partners, *Pms1p/Mlh2p/Mlh3p*, increases SSA [27]. In mammalian cells, MutS homologues *MSH2* and *MSH6* can suppress HeR [24, 28–31], but the role of mammalian MutL homologues in mechanisms inhibiting HeR is less well characterized. Furthermore, whether or not the overall increased HeR and SSA rates from loss of MMR contribute to tumorigenesis is poorly understood.

In tumors, DSBs also occur frequently at chromosomal fragile sites (CFS). CFS are AT-rich repetitive sequences that are difficult-to-replicate during mitosis and manifest as gaps flanked by DSBs in different tumors [32–35]. Fragile sites *FRA16D* and *FRA3B* have increased DSB rates and are the most common CFS reported in tumors [36–39]. *FRA3B* and *FRA16D* are evolutionarily conserved across eukaryotes [36] and encode the tumor suppressors

FHIT and WWOX, respectively. Fragile histidine triad protein (FHIT) (also called bis(5′-adenosyl)-triphosphatase) spans 10 exons over a 1Mb region at fragile site FRA3B. FHIT is a member of histidine triad subfamily of nucleotide binding proteins. FHIT is a DNA hydrolase that metabolizes substrate AP3A (diadenosine 5-prime,5-triple prime-P(1),P(3)-triphosphate), which promotes DNA replication and inhibits stress response signaling [40]. CRCs and gastric carcinomas with MMR deficiency frequently also carry large deletions in FHIT [41]. FHIT deletions also frequently occur in lung cancer [36, 42] *Fhit* knockout mice develop gastric carcinomas and skin cancers [43, 44].

WWOX is a WW domain-containing oxidoreductase at FRA16D. WWOX contains 9 exons and spans 1.2 Mb [43]. Genomic deletions and other structural variants inactivating WWOX occur in almost one-third of solid tumors [45, 46]. WWOX suppresses TGFβ/SMAD signaling [46], and WWOX mouse mutants promote mammary tumor growth [36]. While loss of MMR in CRCs has been associated with increased frequency of FHIT [47] and WWOX [48] deletions, inactivation of FHIT and WWOX at *FRA3B* and *FRA16D* CFS has not been linked to HeR. Here, we report that mammalian MutL homologs *MLH1, PMS2* and *MLH3* participate in suppression of HeR and SSA. In addition to GFP reporter studies, we computationally identified 6,848 paired DRs with 97%–99.9% sequence identity mapping to unique locations in the mouse genome as substrates for endogenous genomic HeR and SSA. Confirming reporter gene studies in yeast, we demonstrate that mutation of *Mlh1, Pms2, Mlh3* or *Mlh3/Pms2* increases HeR in MEF DR sequences. Furthermore, for the 1–12 heterologous bases in each paired DR, we show that >94% of base substitution mutations are consistent with gene conversion (where a mismatched base in one direct repeat motif is replaced with its respective paired direct repeat heterologous base). Next, to assess SSA, we used gamma-irradiation to promote endogenous DSBs and show that *Mlh1*, *Pms2* and *Mlh3* mutations suppress CNAs, including recurrent CNA hotspots that we previously identified in mouse dMMR intestinal tumors. Finally, we analyze data from dMMR and MMR proficient (pMMR) CRCs with matched normal tissues from The Cancer Genome Atlas (TCGA) (http://cancergenome.nih.gov). These studies confirm high rates of human endogenous paired DR HeR and reveal that the intervening sequences between paired DRs are deleted more frequently in dMMR vs pMMR CRCs. Specifically, these dMMR recurrent CNA deletions include hotspots at chromosomal fragile sites *FRA3B* and *FRA16D*, which have flanking DR and IR sequences and high rates of spontaneous DSBs in CRCs. Overall, these data provide insights into mammalian MutL homolog inhibition of HeR and SSA and are consistent with a potential role for dMMR inhibition of homeologous recombination in tumor suppression.

## RESULTS

### Mlh1, Pms2 and Mlh3 suppress direct repeat motif reporter gene homeologous recombination

To understand the role of mammalian MutL homologues in HR and HeR, we modified the pDR-GFP homologous recombination reporter by introducing eight closely spaced mutations in GFP to monitor homeologous recombination, similar to neomycin-based reporters previously used to study the role of mammalian MSH2 in HeR [28] (Supplementary Figure 1). These reporters have 5′ and 3′ overlapping fragments of GFP in the same orientation with intervening puromycin selection cassette and a double stranded nuclease (I-SceI) binding site between the direct repeats. DR-GFP has 100% identity between overlapping sequences and DR-GFP8mu has 8 point mutations (1.2% heterology). Upon transient expression of I-SceI, a double strand break is induced in cells. DR-GFP and DR-GFP8mu (or pneo-Wt and pneo-8 mu) can be repaired by HR/HeR or SSA and scored as GFP+ or GFP- expressing cells by flow cytometry or neomycin positive cells in colony selection assay. We used these reporters for exploratory experiments in *wild-type* (*Wt*), *Mlh1−/−, Pms2−/−, Mlh3−/−, and Pms2−/−;Mlh3−/−* mouse embryonic fibroblasts (MEFs) matched for passage number.

In Wt MEFs transfected with I-SceI, comparisons of DR-GFP and DR-GFP8mu stable transfectants showed that 1.2% heterology suppressed HeR vs HR by 74 ± 3%, whereas *MLH1−/−* MEFs showed only 3 ± 4% suppression (Supplementary Figure 2) (*P* = 0.005, ANOVA followed by Dunnet's test). Further experiments in *Mlh3−/−, Pms2−/−* and *Pms2−/−;Mlh3−/−* MEFs showed 77.5 ± 0.5%, 60 ± 5% and 13.5 ± 0.5% HeR suppression relative to HR (all *p* < 0.001, ANOVA followed by Dunnet's test). These studies are consistent with a potential role for mouse Mlh1 in HeR suppression. Similar to previous findings in IDL repair and DNA damage response, these data also are consistent with partially redundant roles for PMS2 and MLH3 in HeR suppression, as results in *Mlh1−/−* MEFs were not significantly different from those in *Mlh3−/−;Pms2−/−* MEFs (*P* = 0.74, ANOVA followed by Dunnet's test).

### Identification of paired direct repeats in the mouse genome

To understand the roles of MutL homologues in HeR suppression of endogenous direct repeat sequences, we computationally identified paired DR motifs in the mouse genome with uniquely mapping locations and characteristics similar to DR-GFP8mu and DR-Neomut8 reporters. Using the Vmatch program [49], we identified 7,863 mouse DR pairs with the following parameters: 5′ and 3′ DR motifs each > 480 bp in the same orientation with 1–12 mismatched pairs (97%–99.9% identity) and intervening sequences ranging from 500bp to 50 kb (Supplementary Figure 3 and Supplementary Table 1). The average length of paired DRs is 720bp, ranging from 500 bp to 22,359 bp (chr5:105039588-105061946 and 105092514-105114872). The average intervening sequence between paired DRs is 2,157 bp, with the largest 49,972 bp (chr15:74830812-74880784). The set of DRs with 97%–99.9% identity covers 5,035,586bp in the mouse genome. There are 1,031 DRs that overlap with protein coding exons. Chromosome 7 has the highest density of DRs (734/Mbp), while chromosome 16 has the lowest (79/Mbp).

### Mouse genome paired direct repeats have high rates of endogenous homeologous recombination and SSA

DSBs that occur between DRs stimulate both HR and SSA [50]. To understand the role of individual MutL homologues in suppression of HeR and SSA, we treated MEFs with γ-irradiation and cultured for two additional passages. Subsequently, we performed targeted capture of DNA from control and irradiated MEFs for the DR sequences from *Mlh1−/−, Pms2−/−, Mlh3−/−, Mlh3−/−;Pms2−/−* and *Wt* MEFs that are all derived from C57B/L6 genetic background and matched for passage number (*n* = 25). Targeted DR sequences were then analyzed on an Illumina HiSeq 2500. After mapping sequence reads to mouse genome (mm9), and filtering DR reads for mapping quality score >23 and sequence read depth >10 reads, we successfully obtained sequence data for 5,119 paired DR for further analysis.

We compared log-transformed and normalized mutation rates for each MEF genotype, both control and irradiated using multiple linear regression. The analysis revealed no systematic difference in mutation rates with irradiation induced DSBs (*P* > 0.15; ANOVA followed by Dunnet's test). Compared with *Wt* MEF DRs, *Mlh3−/−, Pms2−/−, Mlh3−/−;Pms2−/−* and *Mlh1−/−* MEF DRs all had significantly higher mutation rates (all *P* < 0.001; ANOVA followed by Dunnet's test) (Table 1).

**Table 1:** DR mutation rates and homologous recombination in dMMR MEFs

MEF Phenotype	Overall DR mutations	Mutations at specific DR sites	Mutations outside specific DR sites	GC consistent mutations at specific DR sites	GC inconsistent mutation at specific DR sites	% of GC-consistent mutations at specific DR sites	GC consistent mutations at two contiguous DR sites
Wt	5.9	118.7	4.1	115.8	2.9	97.55686605	8.7
Irradiated Wt	7.1	99.9	5.6	96.9	2.9	96.996997	8.8
Mlh3−/−	41.6	1000.4	25.9	961.3	39.1	96.09156337	58.7
Irradiated Mlh3−/−	42.5	1015.1	26.7	976.2	38.9	96.16786523	55.1
Pms2 −/−	43.1	959.5	28	907.2	52.3	94.5492444	64.6
Irradiated Pms2 −/−	42.4	953.4	27.5	904.1	49.4	94.82903293	58.6
Mlh3 −/− ; Pms2 −/−	44.1	1021.7	28.1	983	38.7	96.21219536	67.7
Irradiated Mlh3 −/− ; Pms2 −/−	45.1	1040	28.9	997.9	42.1	95.95192308	77.8
Mlh1 −/−	61.2	1370.3	40	1306.8	63.5	95.36597825	118
Irradiated Mlh1 −/−	67.1	1574.1	42.5	1523.5	50.7	96.78546471	136.2

Note: For all above, mutation rates were calculated per 10000 bp.

**Figure 1:**
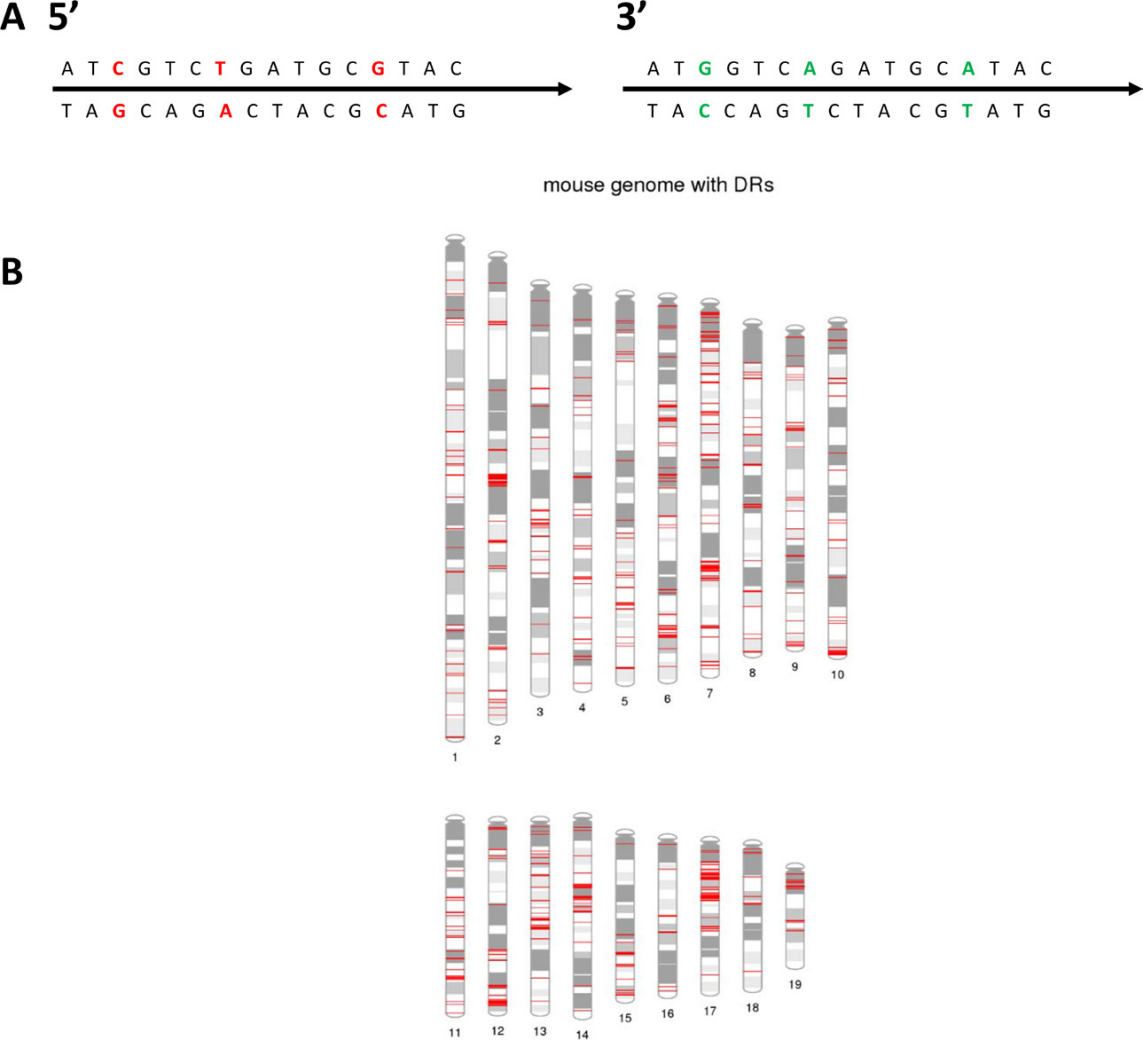
Overview of mouse genome direct repeat sequences (**A**) Schematic illustration of part of a 5′ and 3′ paired direct repeat (DR) sequence with 3 non-identical basepairs that can be monitored for HeR. Non-identical basepairs are shown in red and green. (**B**) Distribution of 5,119 paired DRs in the mouse genome. Red indicates location of a paired DR. Also see Supplementary Figure 3 for the distribution of DRs for each chromosome for mouse and human genomes. Figure was generated using PhenoGram1 [73].

Next, we separately compared mutations at heterologous bases that differed between 5′ and 3′ regions of paired DRs (HeR-BP) and surrounding bases (non-HeR-BP) in DRs with no CNAs. HeR-BPs in DR sequences had significantly higher mutation rates than surrounding bases (Table 1) ranging from 18 to 40 fold higher respectively (all *P* < 0.001; ANOVA followed by Dunnet's Test). In both Wt and dMMR MEFs, for mutations occurring at HeR-BPs, more than 94% of changes were at the corresponding base in the paired DR (e.g. if paired 5′ and 3′ DR HeR-BP sequences are gggCtaa and gggTtaa respectively, the corresponding heterologous mutation in the 3′DR consistent with gene conversion would be gggCtaa) (Figure 1).

The percentage of DR HeR-BP mutations consistent with gene conversion is significantly higher than chance (Table 1; Supplementary Table 1) for all MEF genotypes analyzed. This observation was consistent in both 5′ to 3′ and 3′ to 5′ direction paired DR sequence gene conversions (all *P* < 0.001, Chi-square). Similarly, for longer gene conversion tracts that include two consecutive heterologous bases with mutations consistent with gene conversion, we observed that *Mlh3, Pms2, Mlh1* or combined *Mlh3/Pms2* mutations also significantly increased the number of tracts vs *Wt* MEFs (Table 1). Overall, among the universe of mouse genome DRs, mutation of *Mlh1, Pms2, Mlh3* or *Mlh3/Pms2* significantly increased HeR-BP mutation rates consistent with gene conversion for mouse genome paired DRs vs Wt MEFs (all P<0.001, ANOVA followed by Dunnet's Test) (Table 1). In conclusion, these data are consistent with high rates of endogenous gene conversion in mouse genome paired DR sequences in both Wt and dMMR MEFs. Individual functional mutations of *Mlh3, Pms2, Mlh3* and *Pms2* or *Mlh1* all further increased rates of mutations significantly, consistent with gene conversion in DRs at HeR-BP, as well as overall mutation rates in non-HeR-BP. Mutation rates at HeR-BP were significantly higher than non-HeR-BP bases in DR sequences. *Mlh1* mutation caused the greatest increase in HeR-BP and non-HeR-BP, but individual *Mlh3, Pms2* or combined *Mlh3* and *Pms2* mutations resulted in similar increases (Table 1).

### Increased DR copy number alterations caused by γ-irradiation induced DSBs in Mlh1, Pms2 and Mlh3 mutant MEFs

**Figure 2:**
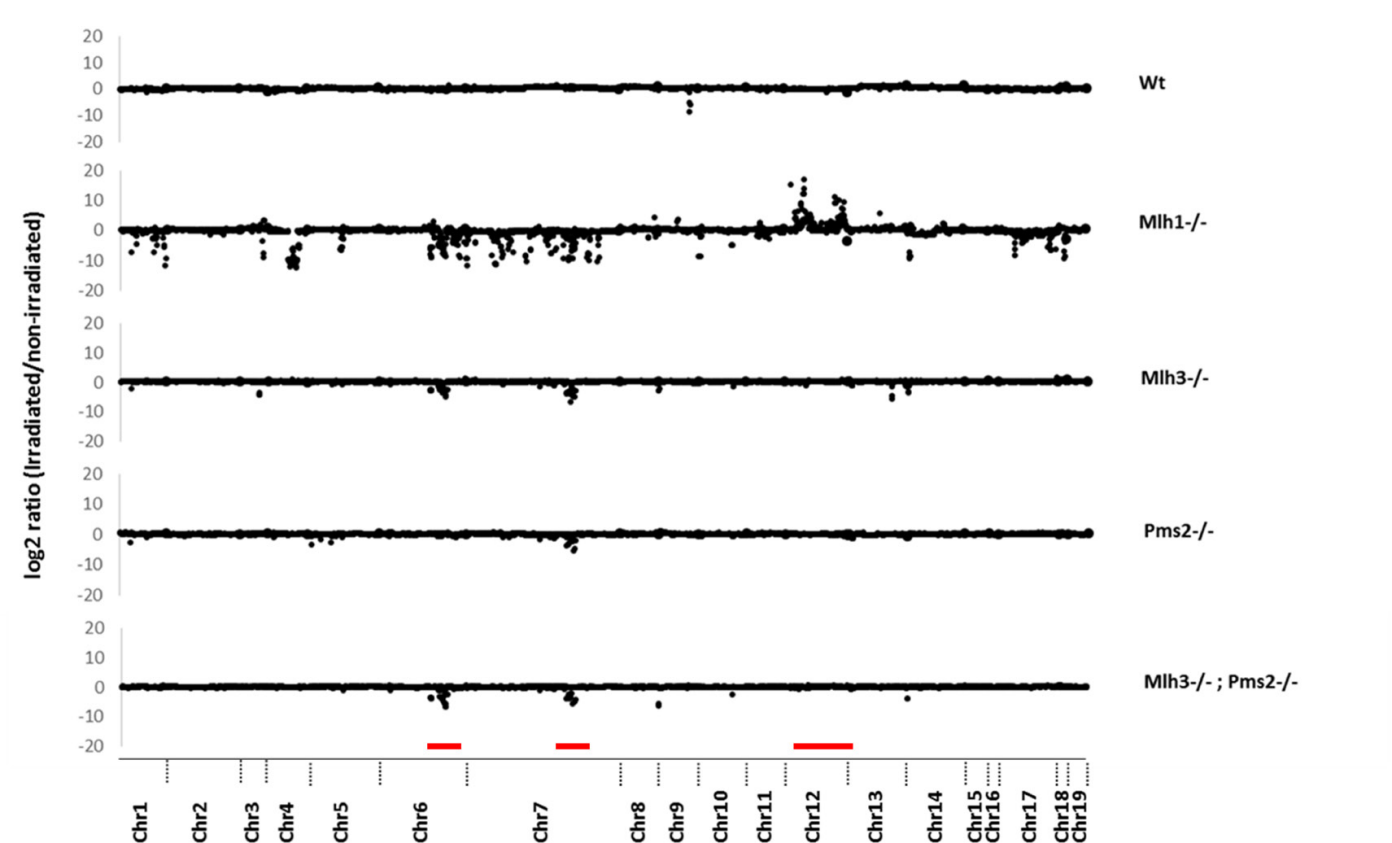
Copy number alterations in direct repeat motifs after irradiation induced double strand breaks Y-axis shows log2 ratio of read depth comparing pre- and post-irradiated DRs. X-axis shows chromosomal location of each DR. MEF genotypes are indicated on the right. Red bars indicate CNAs in chromosome 6, 7 (deletions) and 12 (duplications).

Previously, we identified dMMR enriched recurrent CNAs in mouse *Mlh3−/−;Pms2−/−* intestinal tumors on chromosomes 7 and 12, which were not present in paired normal mucosa [10]. To understand the role of different mammalian MutL homologues in suppression of mouse genomic DR SSA and CNA generation, we compared paired DR sequence read depth of control to γ-irradiated MEFs. *Mlh3−/−* and *Pms2−/−* MEFs had significantly increased DR CNA rates, predominantly deletions, vs *Wt* MEFs (*P* = 1.08 × 10^−12^ and *P* = 0.0203; Chi-square), as did *Mlh3−/−Pms2−/−* and *Mlh1−/−* MEFs (*P* = 5.91 × 10^−13^ and 2.22 × 10^−115^) (Figure 2; Supplementary Table 3). Importantly, *Mlh1−/−* MEFs had overall increased CNA rates vs all other MutL deficient MEFs tested, including *Mlh3−/−;Pms2−/−* (all *p* < 1.09 × 10^−6^; ANOVA followed by Dunnet's Test). Strikingly, shared DR deletion hotspots were observed on mouse chromosomes 6 and 7 (chr6: 89127070-114500739 and chr7: 3957350-3958737; Figure 2). The chromosome 6 CNA deletion hotspot was observed in *Mlh3−/−*, *Mlh3−/−*;*Pms2−/−* and *Mlh1−/−* MEFs, but not *Pms2* mutants, whereas the chromosome 7 CNA deletion cluster hotspot was observed in all MutL mutant MEFs tested (Figure 2). Additionally, in *Mlh1−/−* MEFs, a DR CNA amplification hotspot was observed on chromosome 12 (chr12: 114392088-117316196) (Figure 2). Cross-comparison of these data with previous genome-wide array comparative genomic hybridization (aCGH) studies of dMMR *Mlh3−/−;Pms2−/−* intestinal tumors vs paired normal mucosa revealed that the observed irradiated MEF CNA chromosome 7 and chromosome 12 hotspots overlapped with previously observed hotspot regions in *Mlh3−/−;Pms2−/−* intestinal tumors [10].

In summary, mutation of *Mlh3, Pms2* or *Mlh1* each significantly increased DR CNA rates in γ-irradiated MEFs. Each MutL mutation caused a bias towards genomic deletions, likely due to increased SSA between homologous DR sequences. *Mlh1−/−* MEFs overall had increased CNA rates vs *Mlh3*, *Pms2* or combined *Mlh3;Pms2* mutations. On chromosome 6, an *Mlh3* mutation dependent CNA deletion hotspot was observed that was also seen in *Mlh1−/−* but not *Pms2−/−* irradiated MEFs. A chromosome 7 CNA deletion hotspot shared by *Mlh3*, *Pms2* and *Mlh1* mutant MEFs was also seen that had been previously identified in *Mlh3−/−;Pms2−/−* deficient intestinal tumors. Furthermore, an *Mlh1* (but not other MutL homologue) mutation dependent chromosome 12 CNA amplification hotspot, which had also been previously identified in *Mlh3−/−;Pms2−/−* intestinal tumors, was observed.

### Human dMMR CRC paired DRs have increased rates of homeologous recombination

Using the same methodology and parameters for analysis as in the mouse genome, we identified 12,379 uniquely mappable DRs in the human genome, each carrying 1-12 heterologous bases per paired DR. We then evaluated whether MMR mutations increased paired DR HeR rates in matched normal mucosa-dMMR patient CRC by analyzing genome sequence data from The Cancer Genome Atlas (TCGA-COAD) [51]. We identified 10 dMMR CRCs with matched normal-tumor whole genome sequence with >30× average sequence coverage, and compared them to 10 consecutive pMMR CRCs in TCGA-COAD for somatic mutations in DRs.

As expected, dMMR CRCs have significantly higher mutation rates in DRs (1.36 × 10^−4^ vs 3.7 × 10^−4^ *p* < 0.001, chi-square test) (Table 2). Similar to our findings in DRs in mouse genome, in the human CRCs HeR-BP in DRs have significantly higher mutation rates than surrounding bases (mean 9.75 fold increase, *p* < 0.001, chi-square test). Secondly, consistent with gene conversion and similar to our findings in mouse MEFs, HeR-BP in TCGA dMMR and pMMR CRCs were found to be significantly enriched for the corresponding base substitution in their matched 5′ or 3′DRs (92.86% in dMMR CRCs and 95.44% in pMMR CRCs respectively, all *p* < 0.001, Chi Square test) (Table 2). HeR-BP mutations consistent with gene conversion were also significantly higher in dMMR vs pMMR CRCs (1.02 × 10^−3^ vs 3.2 × 10^−4^ respectively, Chi Square test). Additionally, dMMR CRCs also had higher rates of tracts consisting of two consecutive heterologous bases both with gene conversion consistent mutations (Table 2).

**Table 2:** DR mutation rates and homologous recombination in dMMR vs pMMR CRCs

	hCRC TCGA sample ID	Overall DR mutations	Mutations at specific DR sites (HeR-BP)	Mutations outside specific DR sites (non-HeR-BP)	GC consistent mutations at specific DR sites (HeR-BP)	GC inconsistent mutation at specific DR sites (HeR-BP)	% of GC-consistent mutations at specific DR sites(HeR-BP)	GC consistent mutations at two contiguous DR sites (HeR-BP)
dMMR	aa-3516	0.112	0.783	0.096	0.7	0.083	89.39974457	0.1056
	aa-3518	0.101	0.524	0.09	0.5	0.024	95.41984733	0
	d5-6540	0.093	0.805	0.076	0.748	0.058	92.91925466	0.1149
	aa-a00r	0.102	0.561	0.09	0.463	0.098	82.5311943	0.119
	aa-a01r	0.101	0.99	0.079	0.99	0	100	0.0359
	a6-6781	0.097	1.082	0.074	1.022	0.059	94.45471349	0.0911
	ad-6964	0.095	0.64	0.082	0.598	0.043	93.4375	0.0289
	az-6601	0.093	0.868	0.075	0.821	0.046	94.58525346	0.1931
	ad-a5ej	0.081	0.664	0.067	0.649	0.015	97.74096386	0
	qg-a5z2	0.078	0.649	0.064	0.62	0.029	95.53158706	0
pMMR	aa-3514	0.027	0.236	0.022	0.236	0	100	0
	aa-3534	0.038	0.387	0.029	0.364	0.023	94.05684755	0
	aa-a01x	0.021	0.249	0.015	0.228	0.021	91.56626506	0
	ag-3593	0.032	0.357	0.023	0.357	0	100	0.0538
	ag-3890	0.028	0.268	0.022	0.251	0.017	93.65671642	0
	aa-3685	0.037	0.218	0.032	0.2	0.018	91.74311927	0
	aa-a02y	0.028	0.229	0.023	0.229	0	100	0
	aa-a01v	0.037	0.339	0.029	0.313	0.026	92.33038348	0
	ad-a5ek	0.027	0.301	0.021	0.286	0.015	95.0166113	0
	qg-a5z1	0.035	0.404	0.026	0.389	0.015	96.28712871	0

Note: For all above, mutation rates were calculated per 10000 bp.

Overall, these human CRC data are consistent with studies in mouse MEFs and DRs in the mouse genome. In human DRs, both HeR-BPs and surrounding bases have overall elevated mutation rates when dMMR is compared to pMMR CRCs. However, HeR-BPs have higher mutation rates than surrounding DR bases, almost all mutations are consistent with a mechanism of gene conversion in both pMMR and dMMR CRCs, including tracts of gene conversion consistent mutations. Moreover, dMMR CRCs have higher HeR rates than pMMR CRCs.

### Human dMMR CRC deletion hotspots are at chromosomal fragile sites encoding FHIT and WWOX

**Figure 3:**
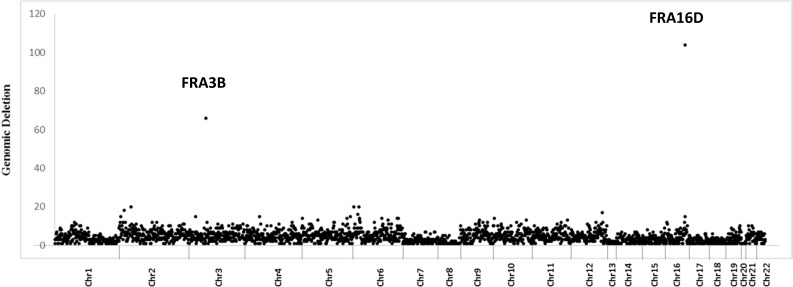
Increased somatic deletions at chromosomal fragile sites FRA3B and FRA16D in dMMR human CRCs Density distribution (number/MB) of deleted probes across all human autosomes in dMMR tumors. FRA3B and FRA16D outliers are indicated.

To understand whether dMMR CRCs have CNA hotspots similar to what we observed in MEFs, we investigated the distribution of genomic regions preferentially deleted only in dMMR CRCs as potential sites for SSA mediated genomic deletions. By comparing CNA deletion rates in 32 dMMR and 156 pMMR tumors, we found that the ratio of CNA deletions in DRs and intervening sequences compared to non-DR sequences is significantly higher in dMMR vs. pMMR tumors (Wilcoxon rank sum test *P* = 0.00947). Of note, this analysis also revealed two dMMR somatic deletion hotspots that mapped to *FRA3B* and *FRA16D* chromosomal fragile sites (CFS) (Figure 3). CFS are long AT-rich repeat genomic sequences that are sites of DNA polymerase stalling during genome replication and have very high rates of spontaneous DSBs in tumors. When DSBs occur at CFS, homologous recombination is known to play an important role in CFS repair. Loci *FRA3B* and *FRA16D*, which encode the tumor suppressor genes FHIT and WWOX respectively, are the two most frequently deleted chromosomal loci in human CRCs. Furthermore, higher rates of FHIT and WWOX deletions have previously been reported in dMMR and pMMR CRC cell lines and tumors [52–58].

**Figure 4:**
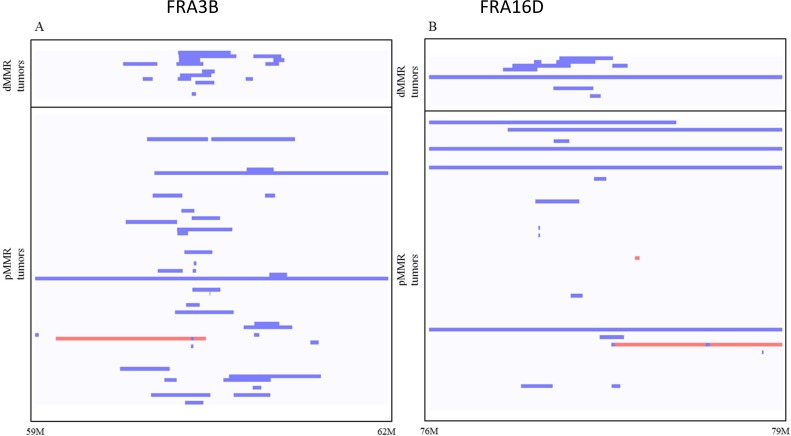
Somatic FRA3B (**A**) and FRA16D (**B**) genomic deletions (blue) and duplications (red) in dMMR and pMMR tumors. Analysis of whole genome sequencing (WGS) data of dMMR and pMMR human CRC tumors from TCGA-COAD. The rates of genomic deletions at FRA3B and FRA16D are significantly higher in dMMR than in pMMR human CRCs (*p* = 0.026 and *p* = 0.017, *t*-test). Blue lines indicate deletion, red lines indicate amplification.

Since *FRA3B* and *FRA16D* common chromosomal fragile sites have high rates of spontaneous DSBs, to understand whether increased SSA recombination between homologous repeat sequences could be a mechanism contributing to increased *FRA3B* and *FRA16D* CNA deletions in dMMR CRCs, we mapped each somatic deletion's flanking breakpoints in genome sequence from 13 TCGA-CoAD dMMR and 77 pMMR CRCs with matched normal tissues and mean sequence coverage >10×. Using Vmatch parameters requiring >80% identity for > 80 bp in length within 1kb of deletion breakpoints, we identified 4 and 0 paired DRs, and 1 and 0 inverted paired DRs respectively in dMMR vs. pMMR CRCs (38.4% vs 0%, *P* = 0.0001 chi-square test). These dMMR CRC genomic deletions were flanked by DR and IR motifs ranging in size from 46 kb to 338 kb (Figure 4; Supplementary Table 4). Notably, these findings are consistent with high rates of spontaneous DSBs at the *FRA3B* and *FRA16D* chromosomal fragile sites in human dMMR CRCs that promote SSA and deletions at the FHIT and WWOX tumor suppressor loci.

## DISCUSSION

DNA mismatch repair plays critical roles in maintaining genome integrity and inhibiting tumorigenesis. For repetitive sequences, suppression of HeR and SSA are important mechanisms to maintain genomic integrity. However, the role of individual mammalian MutL homologues in preserving genomic integrity by suppressing HeR and SSA between similar DNA sequences is poorly characterized. Furthermore, the roles of HeR and SSA in tumor suppression are unclear.

Building upon previous studies with HeR reporter genes [28], we demonstrate that genomic DRs can serve as *in vivo* endogenous reporters for measuring the levels of suppression or induction of HeR and SSA. In Wt MEFs, endogenous mutations in DRs are dominated by gene conversion, with HeR-BP mutation rates 33–40 fold higher than surrounding bases and >94% consistent with gene conversion from the corresponding DR paired sequence. Loss of function mutations in *Mlh1, Pms2, Mlh3* and combined loss of function mutations in *Mlh3;Pms2* further increase rates of mutations significantly, which is consistent with gene conversion in DRs at HeR-BP, as well as overall mutation rates in non-HeR-BP. Using both GFP reporter and genomic DRs, we extend previous reporter gene based studies of *Saccharomyces cerevisiae Mlh1p* and *Pms1p* (ortholog of *Pms2*) [23–26] to mammalian cells and show that mouse MutL homologs *Mlh1* and *Pms2* are involved in HeR suppression. Furthermore, we extend these findings and provide evidence for mammalian *Mlh3* involvement in HeR suppression as well (summarized in Supplementary Figure 4A).

We evaluated DR mutations in human dMMR and pMMR CRCs by using available whole genome sequencing data from the TCGA database (http://cancergenome.nih.gov). In human genome DRs, HeR-BPs have higher mutation rates than surrounding DR bases. Almost all mutations are consistent with a mechanism of gene conversion in both pMMR and dMMR CRCs, including tracts of two consecutive gene conversion consistent mutations. Overall, these findings are largely consistent with MEF studies, supporting DRs as *in vivo* endogenous HeR reporters and demonstrating that dMMR CRCs have higher HeR rates than pMMR CRCs.

Next, using γ-irradiation to induce DSBs, we show that DR paired sequences can also be used to monitor SSA. Consistent with *Saccharomyces cerevisiae Mlh1p* reporter gene studies [27], loss of function mutations in *Mlh1, Pms2* or *Mlh3* each significantly increased DR CNA rates (summarized in Supplementary Figure 4B).

For all mutant MEFs, there was a bias towards DR genomic deletions, which is most likely due to increased SSA between homologous DR sequences. Similarly, analysis of TCGA data also support increased rates of SSA in dMMR compared to pMMR in human CRCs. MMR deficiency increased the rate of CNA deletions for both DRs and the intervening sequences between matching DR pairs.

Interestingly, analysis of MEF showed that Mlh1−/− cells have significantly higher overall CNA rates compared to Pms2−/−, Mlh3−/− or even Mlh3−/−;Pms2−/− MEFs. While we did not have Pms2−/−;Mlh3−/−;Pms1 triple mutant MEFs to study, given that Mlh1-Pms2, Mlh1-Mlh3 and Mlh1-Pms1 constitute all known MutL complexes, our data suggest a potential previously unexplored role for the Mlh1-Pms1 MutL complex in suppression of SSA.

Unexpectedly, in both mouse MEF and human TCGA studies, we identified evidence of specific CNA hotspots. For MEF with DSBs induced by gamma irradiation, hotspots included (a) a mouse chromosome 6 deletion hotspot shared by Mlh1−/−, Pms2−/−, Mlh3−/− and Mlh3−/−;Pms2−/− MEFs, (b) mouse chromosome 7 deletion hotspot shared by Mlh1−/−, Pms2−/−, Mlh3−/− and Mlh3−/−;Pms2−/− MEFs that we had been previously identified in *Mlh3−/−;Pms2−/−* deficient intestinal adenocarcinomas but not matched normal mucosa or Mlh3−/− adenomas [10] and (c) a mouse chromosome 12 amplification hotspot in Mlh1−/− MEFs, also previously seen in *Mlh3−/−;Pms2−/−* deficient intestinal adenocarcinomas [10]. Each of these regions is rich in highly repetitive elements. The mouse chromosome 6 deletion-hotspot occurs in a gene desert with no obvious unique feature, while the chromosome 7 hotspot contains a cluster of highly homologous paired immunoglobulin receptor (PiR) genes and the chromosome 12 hotspot contains many Immunglobulin Heavy chain (IgH) genes with a high degree of homology. We propose that mouse PiR and IgH gene clusters act as endogenous substrates for homeologous DR and inverted repeat (IR) formation. For chromosome 6 and 7 loci, the predominance of DR deletions is consistent with MutL suppression of SSA. However, for the chromosome 12 hotspot, there is a strong bias of CNAs to cause copy number gains. The precise mechanism of recombination at this latter locus and the precise features of each loci that specifically cause great dependence on MMR to suppress recombination are unclear at this time.

In human CRCs, analysis of DRs and IRs revealed two somatic deletion dMMR hotspots at chromosomal fragile sites (CFS) *FRA3B* (chr3:58,600,000–63,700,000) and *FRA16D* (chr16:79,200,000–81,700,000). CFS are long AT-rich repeat genomic sequences that are sites of DNA polymerase stalling during genome replication and have very high rates of spontaneous DSBs in tumors [59–61]. When DSBs occur at CFS, homologous recombination is known to play an important role in CFS repair [62–65]. *FRA3B* and *FRA16D*, which encode the tumor suppressor genes FHIT and WWOX respectively, are the two most frequently deleted chromosomal loci in human CRCs. Higher rates of FHIT and WWOX deletions have previously been reported in dMMR vs pMMR CRC cell lines and tumors [66, 67]. Mapping revealed in dMMR CRCs higher rates of DR and IR motifs in breakpoints flanking *FRA3B* and *FRA16D*. Since CFS have high endogenous rates of DSBs, our data are consistent with increased rates of SSA promoting deletions of FHIT and WWOX tumor suppressors in dMMR tumor.

In summary, known DNA mismatch repair tumor suppression mechanisms include base-substitution and small in/del mutation repairs as well as DNA damage response initiation. Our data provide the first evidence that homeologous recombination and SSA suppression are also important MMR tumor suppression mechanisms. Poly ADP (Adenosine Diphosphate)-Ribose Polymerase (PARP) inhibitors are an example of a successful mechanistically based therapy that exploits synthetic lethality with HR deficiency in ovarian and breast tumors. Our findings suggest that screens to identify drugs promoting synthetic lethality with increased HeR rates may similarly be effective for targeted treatment of dMMR tumors.

## MATERIALS AND METHODS

### Mouse embryonic fibroblast cell culture

Mouse embryonic fibroblasts were established from day 12.5 embryos isolated from the uteri of pregnant mice and matched for passage number as previously described [11]. In brief embryos were digested in Trypsin-EDTA 10 minutes in an incubator (37°C; 5% CO_2_) and washed with PBS. MEFS were then grown in 15% FBS and 100U/ml penicillin, 100 ug/ml streptomycin at 37C, 5% CO2.

### Mouse embryonic fibroblast transfection

We constructed pDR-GFP8mu containing 8 silent mutations in the iGFP of pDR-GFP [28]. Primary MEFs were transfected with either pDR-GFP or pDR-GFP8mu and pCβASc by electroporation using a Nucleofactor device. Briefly, 2 × 10^7^ cells in 1 ml of phosphate-buffered saline were electroporated with 20 to 25 μg of each uncut plasmid DNA in a 0.4-cm electrode-gap cuvette (250 V, 960 μF). Electroporated cells were aliquoted into four or five 10-cm-diameter dishes and kit for primary cells. As I-*Sce*I is expressed and recombinant cells expressing GFP were analyzed using flow cytometry (BD-Biosciences) or colonies were selected in media 24 h after electroporation and were grown in selection G418 (200 μg/ml) media for 14 days before colony counts.

### MEF gamma irradiation

Primary MEFS from all cell lines were cultured and irradiated with 5 Grays @ 1.27 Gy/Min. Subsequently the cells were fed fresh media and allowed to grow and passaged twice. The cells were then harvested and genomic DNA was extracted using DNeasy kit (Qiagen) per manufacturer's protocol. All experiments were performed by irradiating exponentially growing cell cultures with 137Cs irradiator (Mark 1 irradiator, JL Shepherd & Associates).

### Direct repeat region identification

To search for direct repeat (DR) regions in mouse genome (mm9) and human genome (hg19) we used the program Vmatch [68] using the following parameters: minimal length of repeat region 480 bp, minimal gap between DR regions 500 bp, and maximal gap 50Kb; minimal sequence identity between paired DRs >97%, and maximum number of non-identical base pairs is 12/DR.

### Sequence analysis

Paired-end reads sequenced at targeted direct repeat regions were processed at Weill Cornell Genomic Core and then mapped to mouse reference genome (mm9) with BWA method [69]. After removing PCR duplicates, only uniquely mapped and properly paired reads were considered for depth calculation. Variants in the direct repeat regions were called with SAMtools software [70]. These variants were then filtered with standard thresholds for quality (q30), depth (50), mapping quality (30), and genotype quality (9\0). Mutation variant “Alt-allele” calls had median 41% mean allele frequency.

### TCGA SNP array analysis

Level 2 and level 3 SNP array data were downloaded from TCGA data portal (http://cancergenome.nih.gov). For each probe, the ratio of normalized probe signal between tumor and the matched normal tissue was used to determine if there was a copy number alteration at the probe site. We considered a corresponding CNA at a site if tumor to normal ratio was >1.5 or <0.5. At each probe site, differential CNA between dMMR and pMMR tumors was calculated with Chi-squared analysis based on the 2 × 2 contingency table. If the expected values in the contingency table were less than 1, we used Fisher exact test following recommendations of Campbell et al. [71].

### TCGA COAD whole genome sequence analysis

For 10 dMMR CRC patients with available whole genome sequencing tumor and matching normal germline DNA data, pre-processed BAM files were downloaded from TCGA database portal (http://cancergenome.nih.gov). In addition, for 10 pMMR CRC patients, tumor and matching normal whole genome sequence data were also downloaded from TCGA and READ portals. The selection of these patients was based on matching sequence depth in addition to same sequencing center, to reduce potential batch effects. Somatic point mutations for each CRC patient were identified using Mutect [72] method by joint calling the TCGA pre-processed tumor and normal BAM files.

## SUPPLEMENTARY MATERIALS FIGURES AND TABLES



## Abbreviations

BP: Base pair
BIR: Break induced replication
CFS: Chromosomal fragile site
CNA: Copy number alteration
CO: Crossover
CRC: Colorectal cancers
dMMR: Deficient DNA mismatch repair
DR: Direct sequences
DSBs: Double-strand breaks
DSBR: Double-strand break repair
HR: Homologous recombination
HeR: Homeologous recombination
IDL: Insertion/deletion loops
pMMR: Proficient DNA mismatch repair
IR: Inverted repeats
ssDNA: Single-stranded DNA
SDSA: Synthesis dependent strand annealing
SSA: Single strand annealing
