# Supplementary material for: Inhibition of colorectal cancer genomic copy number alterations and chromosomal fragile site tumor suppressor FHIT and WWOX deletions by DNA mismatch repair
Source: Oncotarget. 2017 May 10;8(42):71574–86. doi: 10.18632/oncotarget.17776 (PMC5641073; doi:10.18632/oncotarget.17776)
Supplement: Supplementary file 1 [file oncotarget-08-71574-s001.pdf]

Inhibition of colorectal cancer genomic copy number alterations and chromosomal fragile site tumor suppressor FHIT and WWOX deletions by DNA mismatch repair

Supplementary Materials

Supplementary Table 1: Mouse Genome 5′DR, Mouse Genome 3′DR. See Supplementary_Table_1

Supplementary Table 2: Non-HeR BP mutation rates in DRs

	A	C	G	T
A		0.26<br>9	0.49<br>6	0.23<br>4
C	0.21<br>1		0.15<br>9	0.61<br>2
G	0.61<br>8	0.19<br>7		0.18<br>6
T	0.22<br>5	0.62<br>4	0.15<br>0	

Supplementary Table 3: Copy number alterations (CNA) in pMMR and dMMR mouse embryonic fibroblasts (MEFs)

Genotype	CNA rate
Wt	0.016
Mlh1–/–	0.198
Mlh3–/–	0.059
Pms2–/–	0.038
Mlh3l–/–;Pms2–/–	0.059

Supplementary Table 4: Direct repeats (DRs) and inverted repeats (IRs) within chromosomal fragile sites *FRA3B* and *FRA16D* flanking deletions in dMMR CRCs

Chromosome	5' start position (bp)	5' end position (bp)	3' start position (bp)	3' end position (bp)	Sequence type	Sequence identity (%)	Sequence length (bp)	Gap (bp)
3	60265517	60265716	60310071	60310270	DR	84.0	200	44355
3	60310138	60310271	60402645	60402778	DR	87.3	134	92374
3	60210601	60210681	60326575	60326655	DR	92.6	81	115894
16	77009170	77009310	77249799	77249939	IR	86.5	141	240489
16	77056537	77056779	77395521	77395763	DR	83.5	243	338742

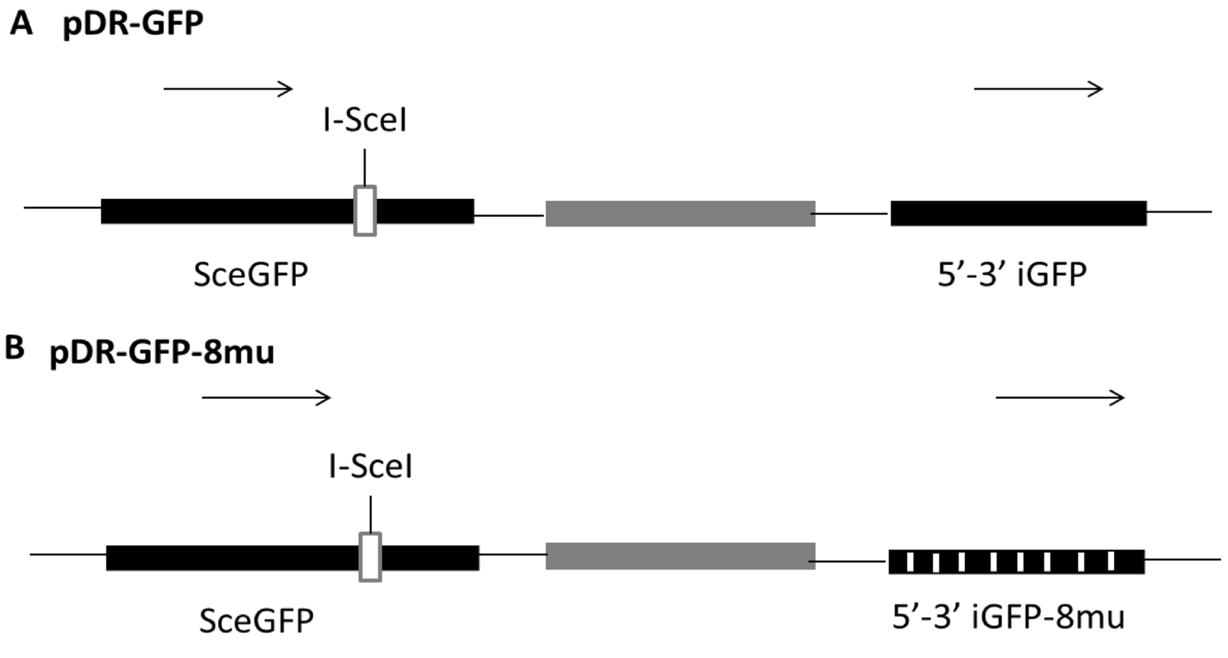

Supplementary Figure 1: HR and HeR GFP expression plasmids. (**A**) In DR–GFP recombination substrate, the GFP is modified to SceGFP, to contain an I-SceI site and in-frame termination codons. Downstream of the SceGFP gene is iGFP, a 5′ and 3′-truncated GFP gene. (**B**) In DR-GFP-8mu, the iGFP-8mu, a 5′ and 3′ containing 8 silent mutations denoted by white bars (1:bp 51 G->C; 2:bp 132 C->A;3:bp 294 G->C; 4:bp 336 G->C; 5:bp 429 C->G; 6:bp 477 C->A; 7:bp 534 C->G; 8:bp 639 C->T).

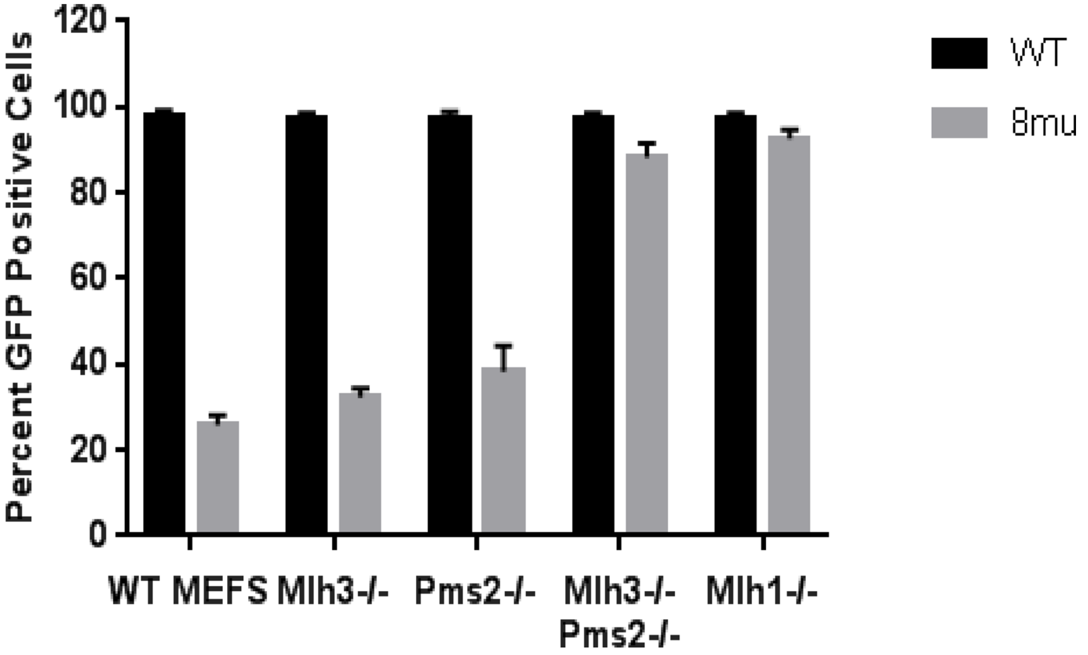

Supplementary Figure 2: Primary MEFs from Wt, Mlh1–/–, Pms2–/–, Mlh3–/–, and Pms2–/–;Mlh3–/– mice stably expressing pDRGFP and pDRGFP8mu were transfected with an I-SceI expression vector and level of recombination was measured by flow cytometry analysis of GFP+ and GFP- cells. All $P < 0.001$, ANOVA followed by Dunnet's test.

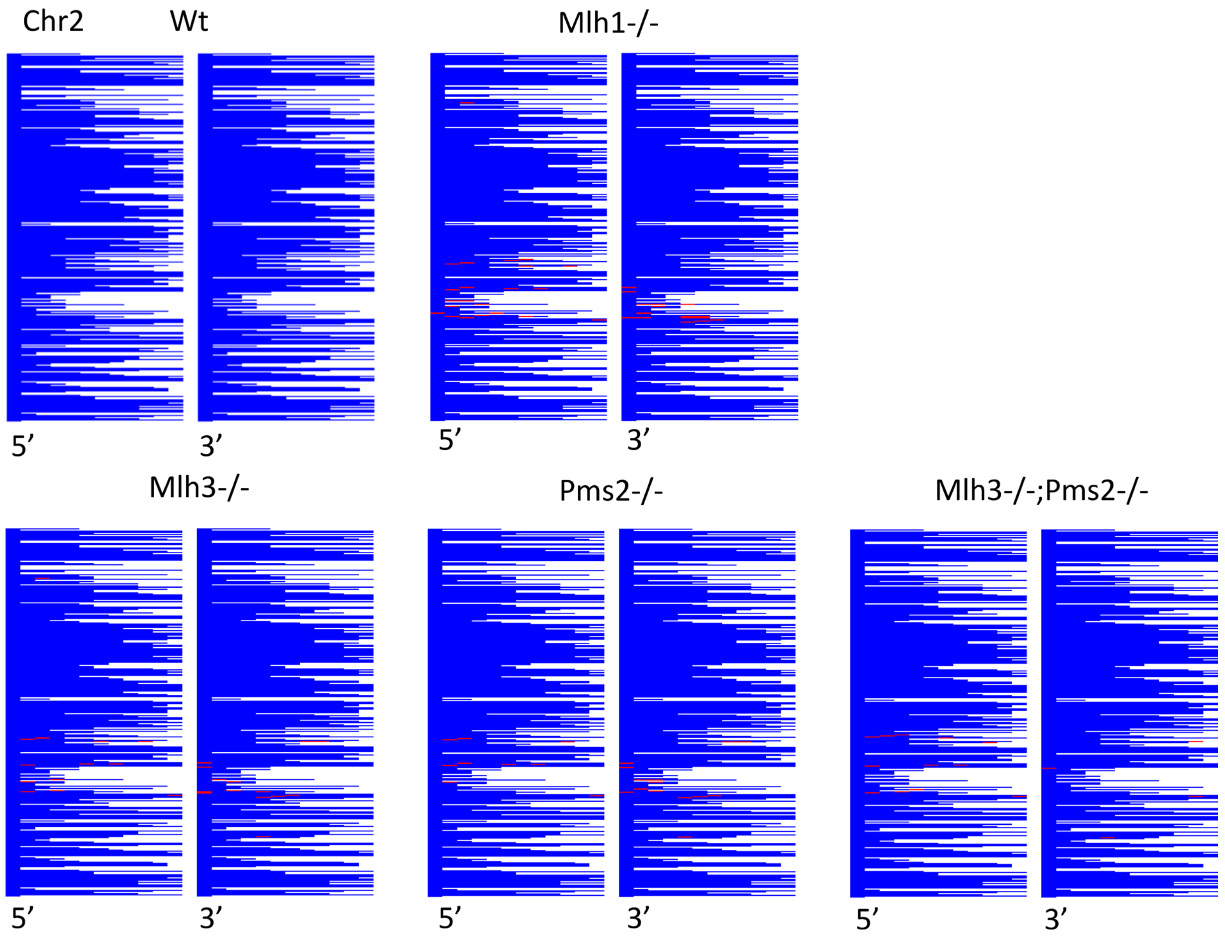

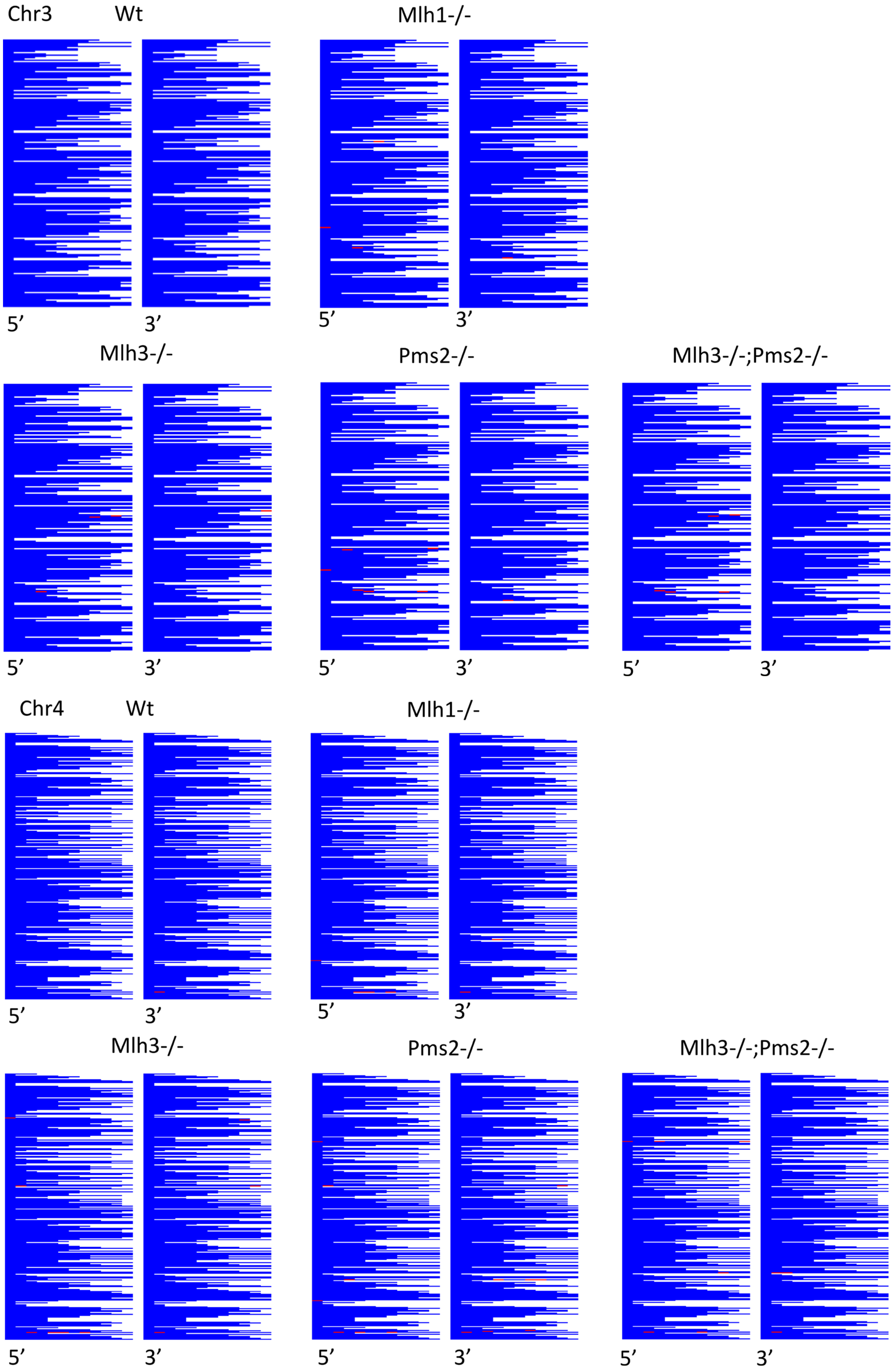
Chr3
Wt
5'
3'
Mlh1-/-
5'
3'
Mlh3-/-
5'
3'
Pms2-/-
5'
3'
Mlh3-/-;Pms2-/-
5'
3'
Chr4
Wt
5'
3'
Mlh1-/-
5'
3'
Mlh3-/-
5'
3'
Pms2-/-
5'
3'
Mlh3-/-;Pms2-/-
5'
3'

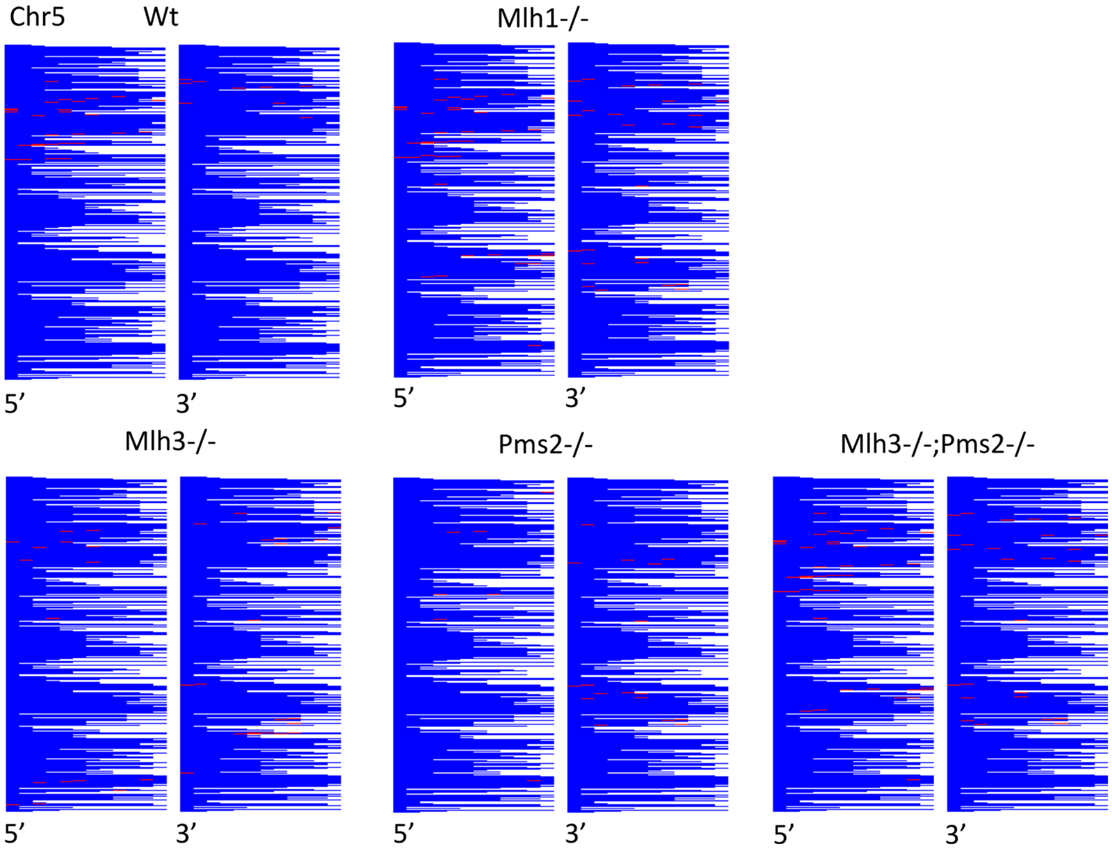
Chr5
Wt
Mlh1-/-
5'
3'
5'
3'
Mlh3-/-
Pms2-/-
Mlh3-/-;Pms2-/-
5'
3'
5'
3'
5'
3'

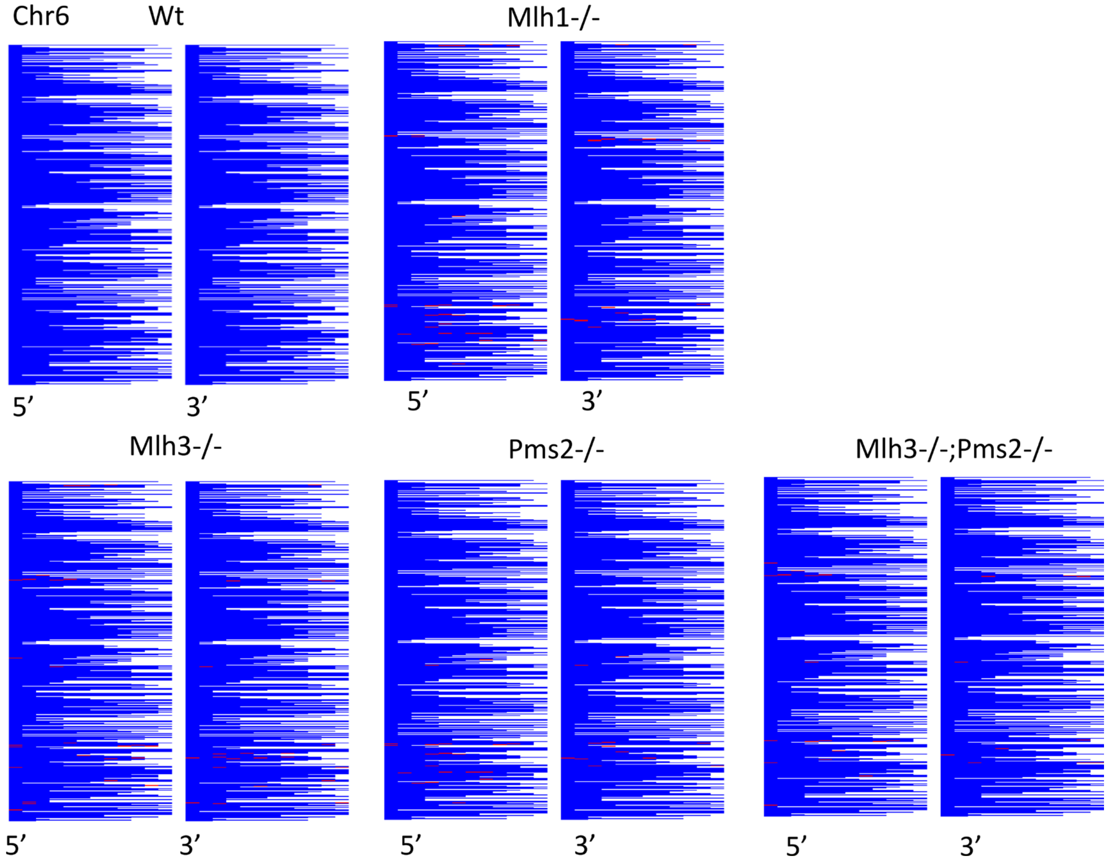
Chr6
Wt
Mlh1-/-
5'
3'
5'
3'
Mlh3-/-
Pms2-/-
Mlh3-/-;Pms2-/-
5'
3'
5'
3'
5'
3'

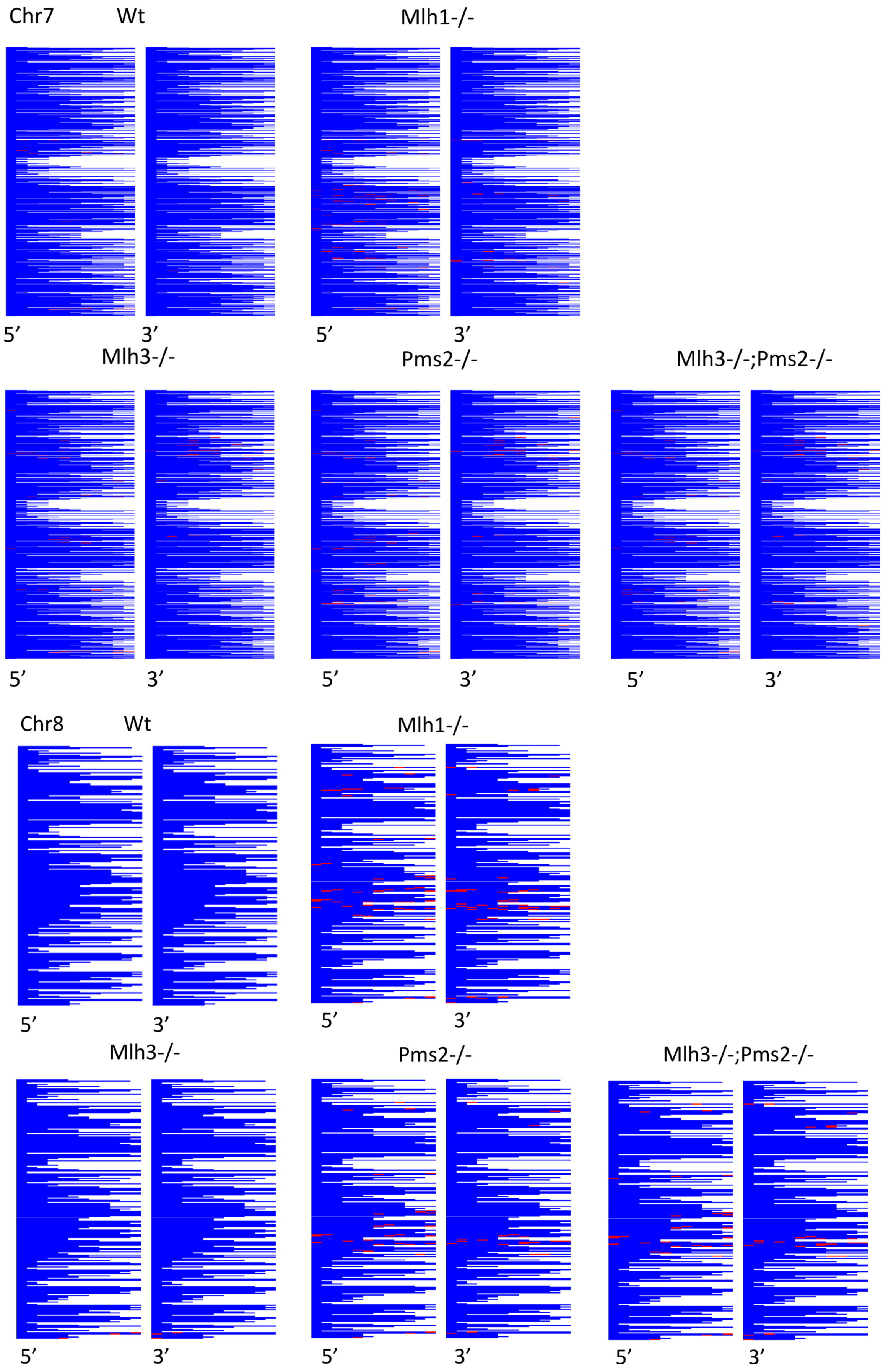
Chr7
Wt
Mlh1-/-
5’
3’
5’
3’
Mlh3-/-
Pms2-/-
Mlh3-/-;Pms2-/-
5’
3’
5’
3’
5’
3’
Chr8
Wt
Mlh1-/-
5’
3’
5’
3’
Mlh3-/-
Pms2-/-
Mlh3-/-;Pms2-/-
5’
3’
5’
3’
5’
3’

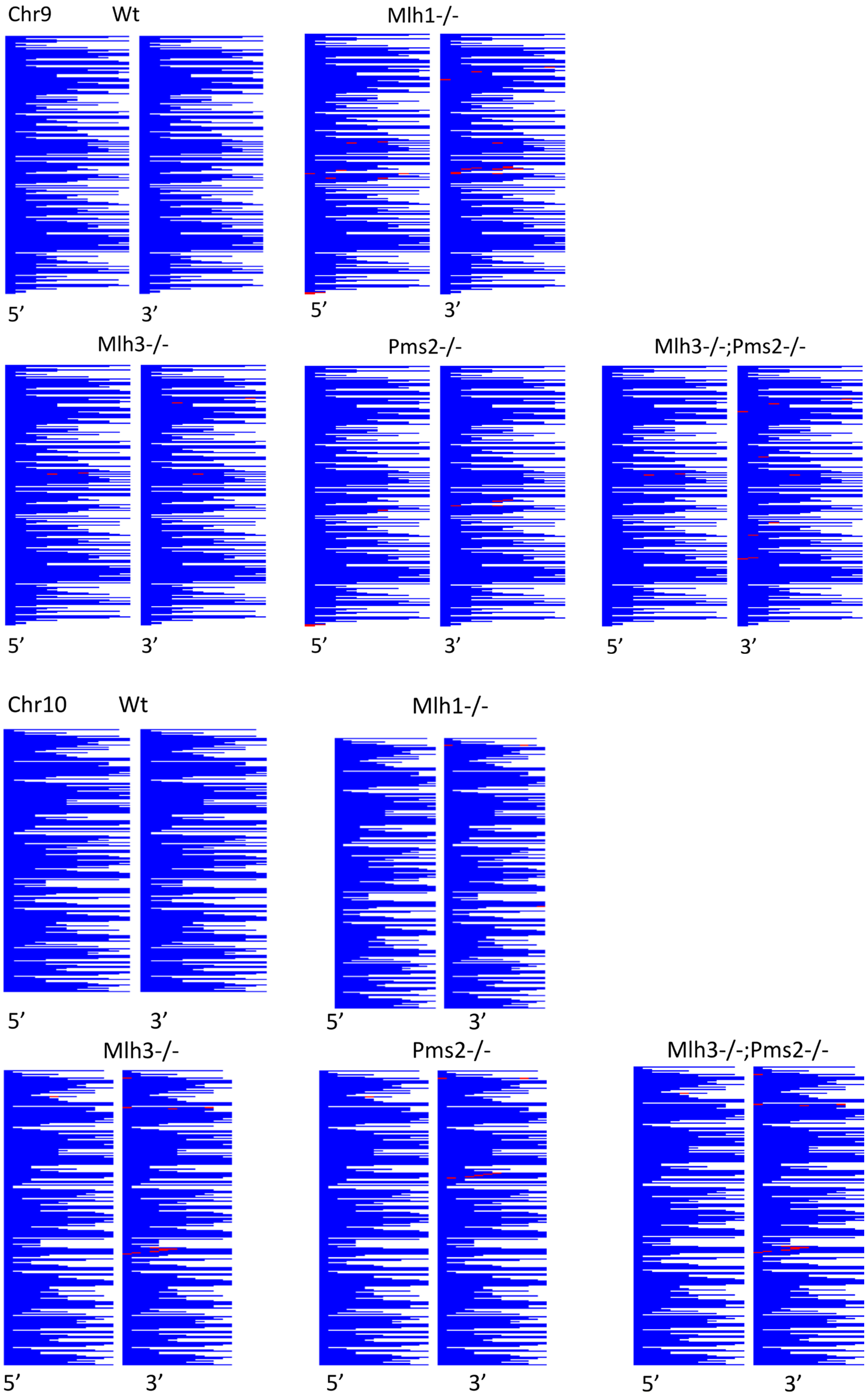
Chr9
Wt
5′
3′
Mlh1-/-
5′
3′
Mlh3-/-
5′
3′
Pms2-/-
5′
3′
Mlh3-/-;Pms2-/-
5′
3′
Chr10
Wt
5′
3′
Mlh1-/-
5′
3′
Mlh3-/-
5′
3′
Pms2-/-
5′
3′
Mlh3-/-;Pms2-/-
5′
3′

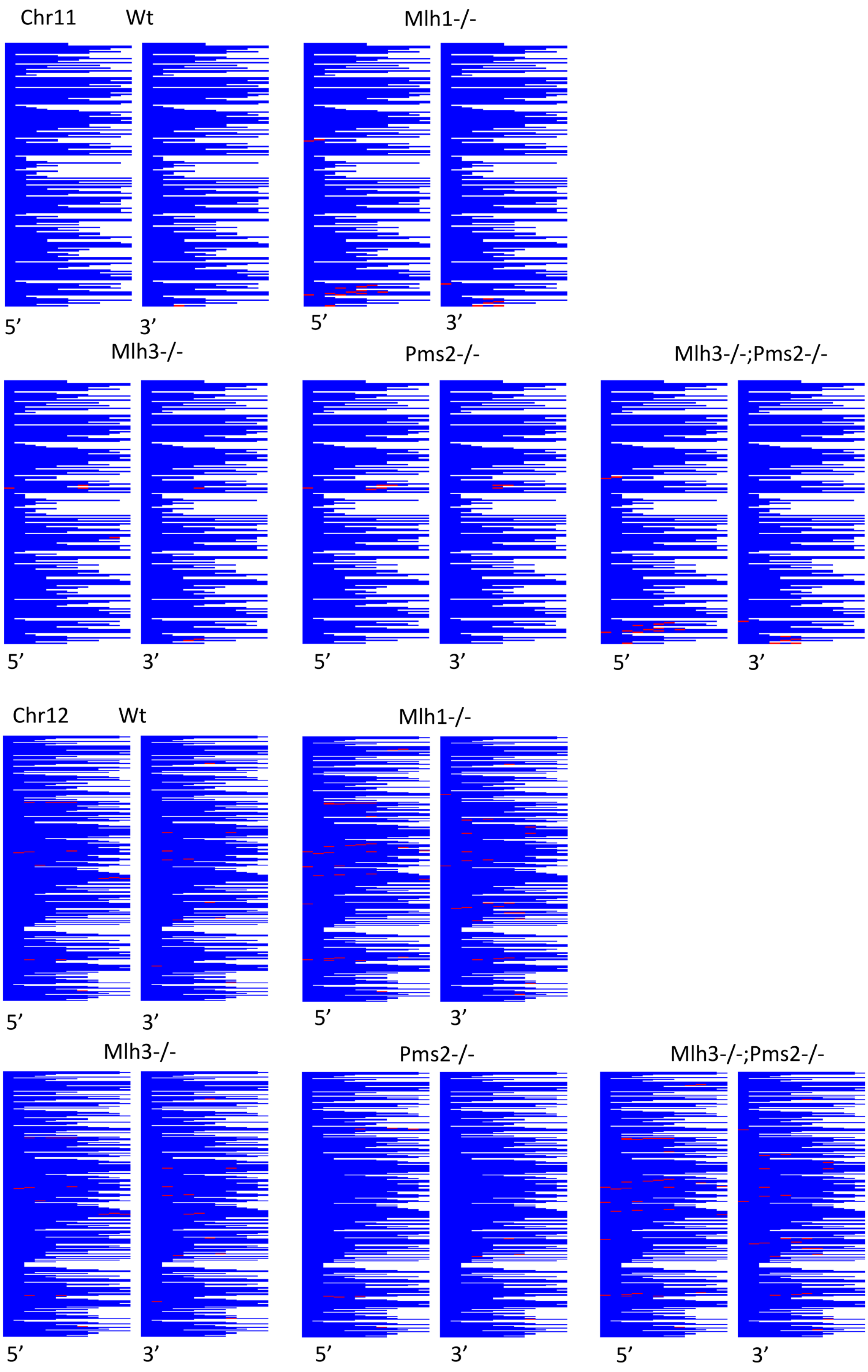
Chr11
Wt
Mlh1-/-
5’
3’
5’
3’
Mlh3-/-
Pms2-/-
Mlh3-/-;Pms2-/-
5’
3’
5’
3’
5’
3’
Chr12
Wt
Mlh1-/-
5’
3’
5’
3’
Mlh3-/-
Pms2-/-
Mlh3-/-;Pms2-/-
5’
3’
5’
3’
5’
3’

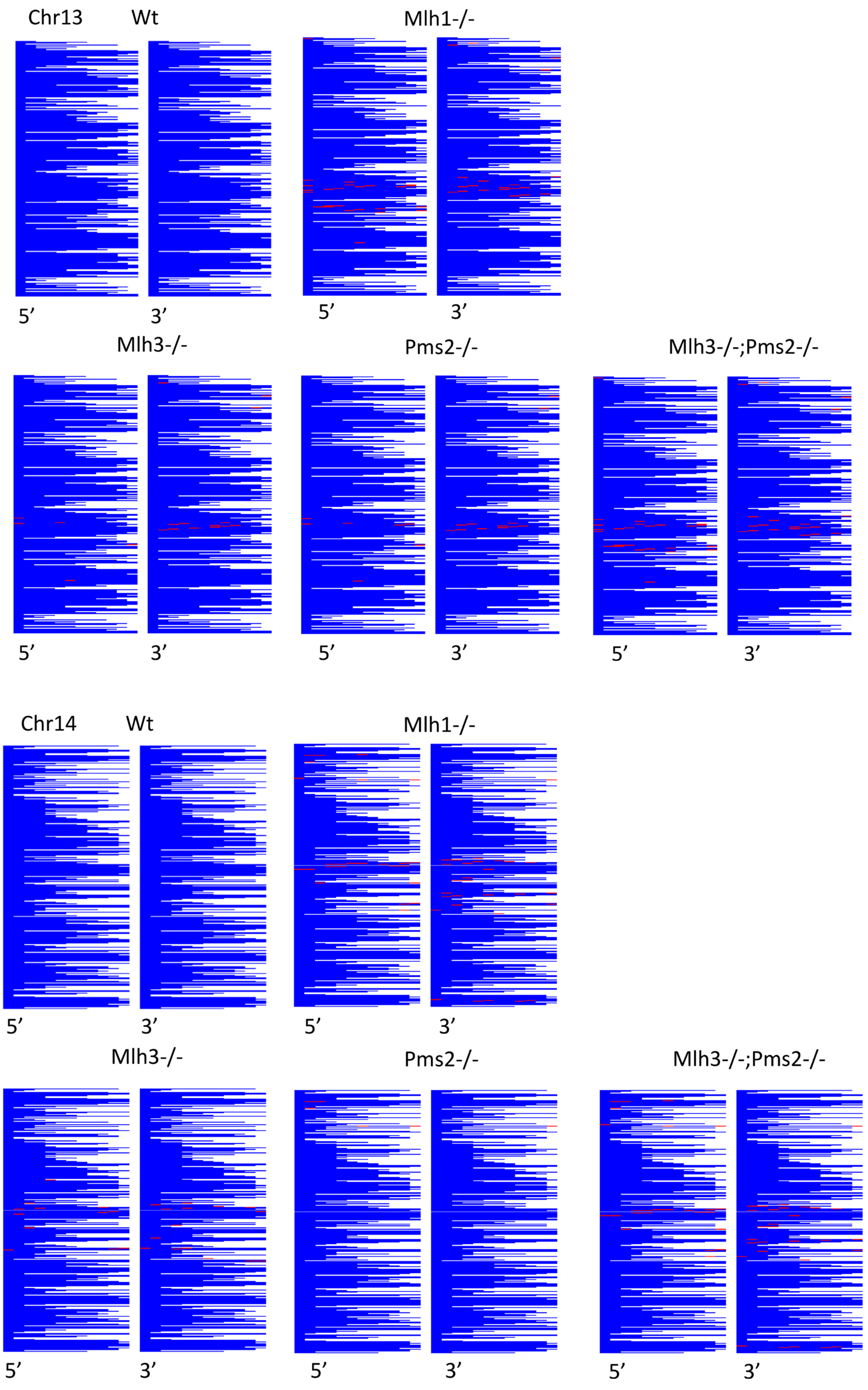
Chr13
Wt
Mlh1-/-
5'
3'
Mlh3-/-
Pms2-/-
Mlh3-/-;Pms2-/-
Chr14

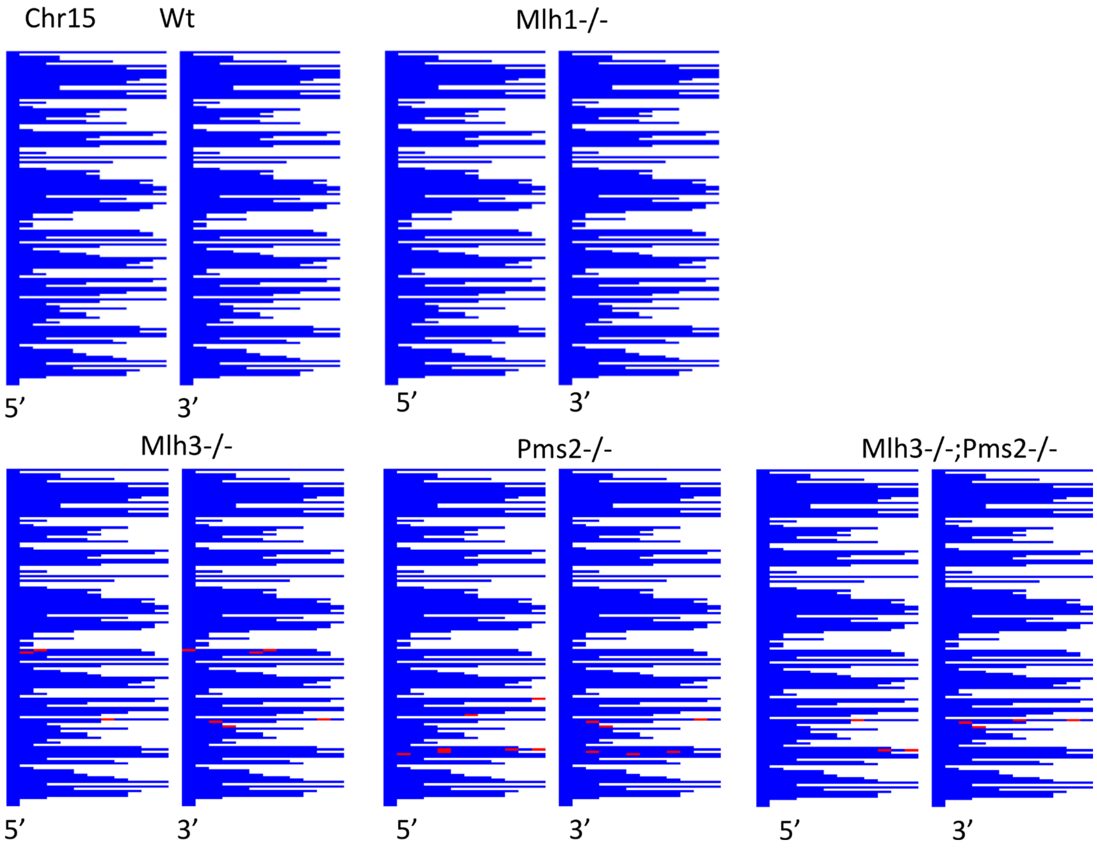
Chr15
Wt
Mlh1-/-
5'
3'
5'
3'
Mlh3-/-
Pms2-/-
Mlh3-/-;Pms2-/-
5'
3'
5'
3'
5'
3'

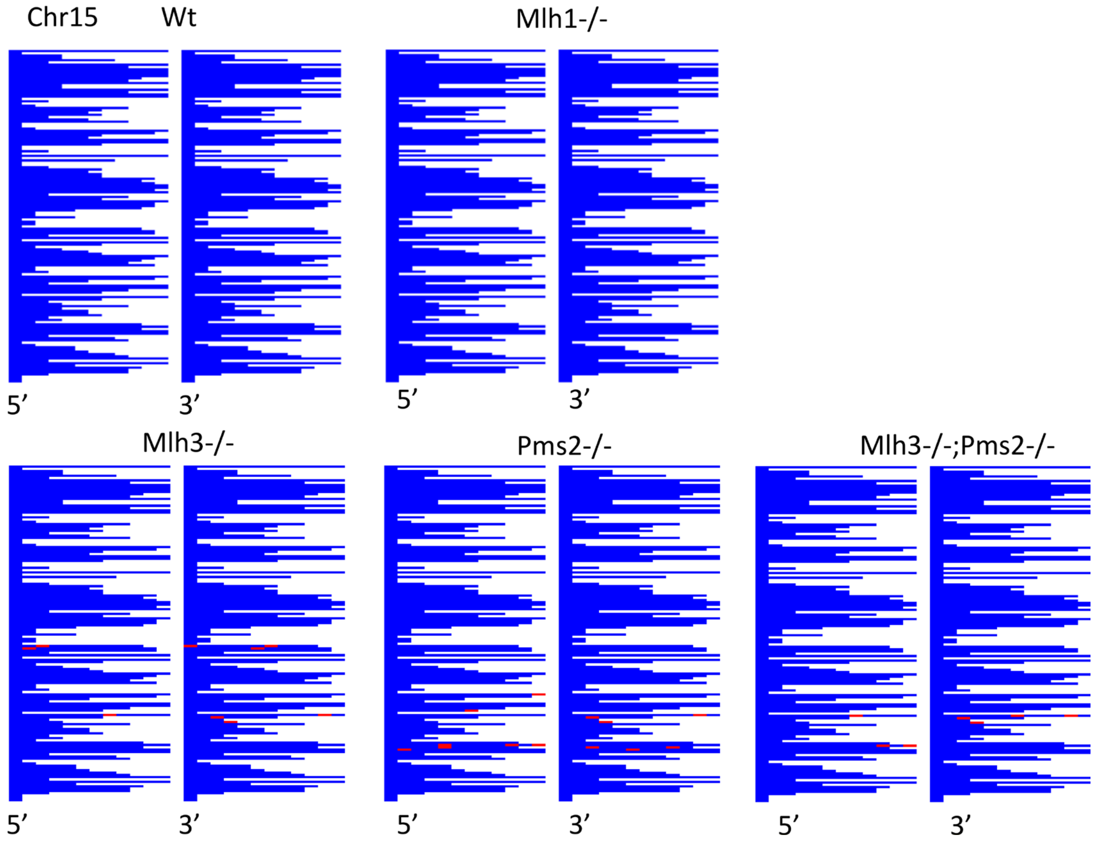
Chr15
Wt
Mlh1-/-
5'
3'
5'
3'
Mlh3-/-
Pms2-/-
Mlh3-/-;Pms2-/-
5'
3'
5'
3'
5'
3'

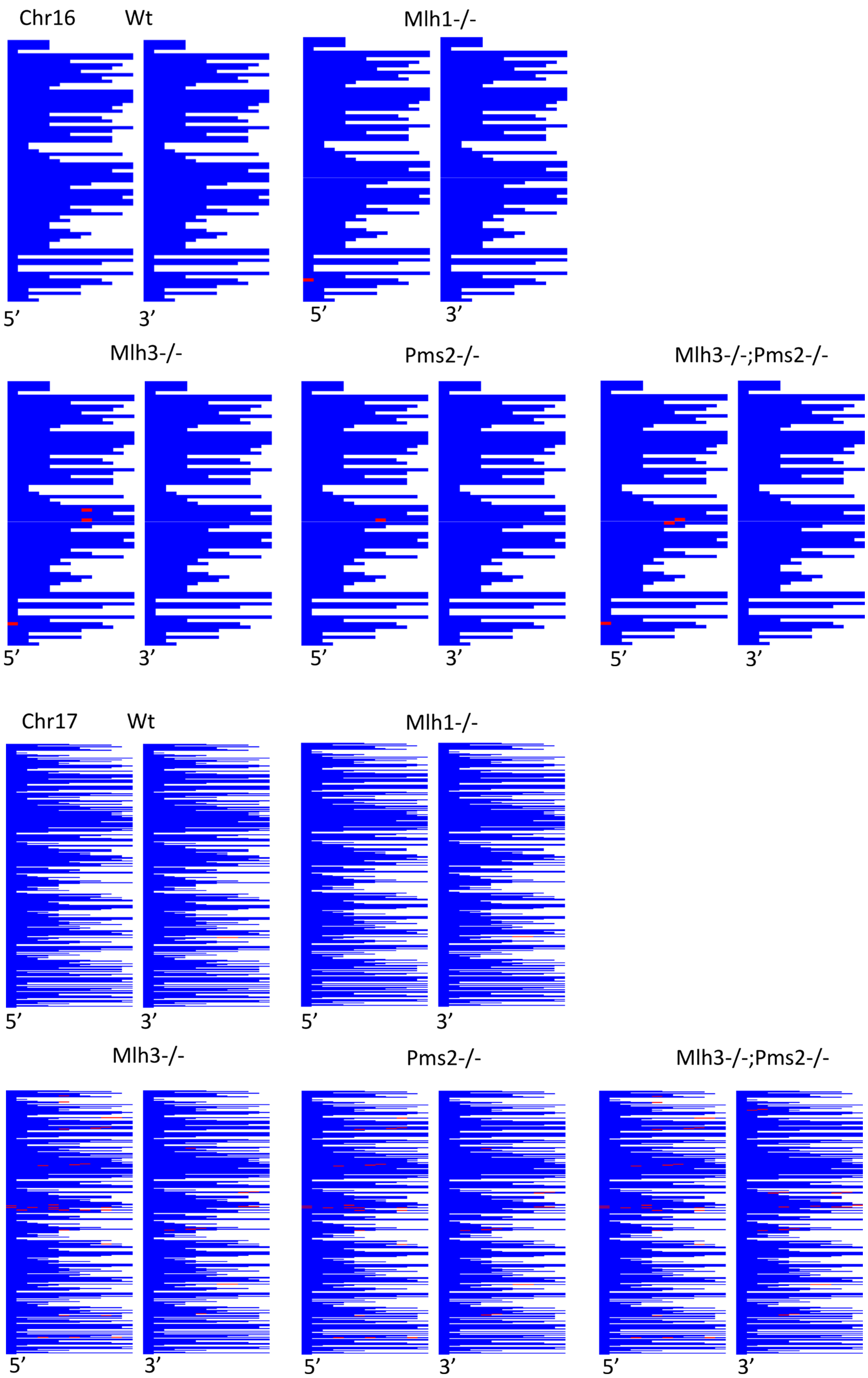
Chr16
Wt
5′
3′
Mlh1-/-
5′
3′
Mlh3-/-
5′
3′
Pms2-/-
5′
3′
Mlh3-/-;Pms2-/-
5′
3′
Chr17
Wt
5′
3′
Mlh1-/-
5′
3′
Mlh3-/-
5′
3′
Pms2-/-
5′
3′
Mlh3-/-;Pms2-/-
5′
3′

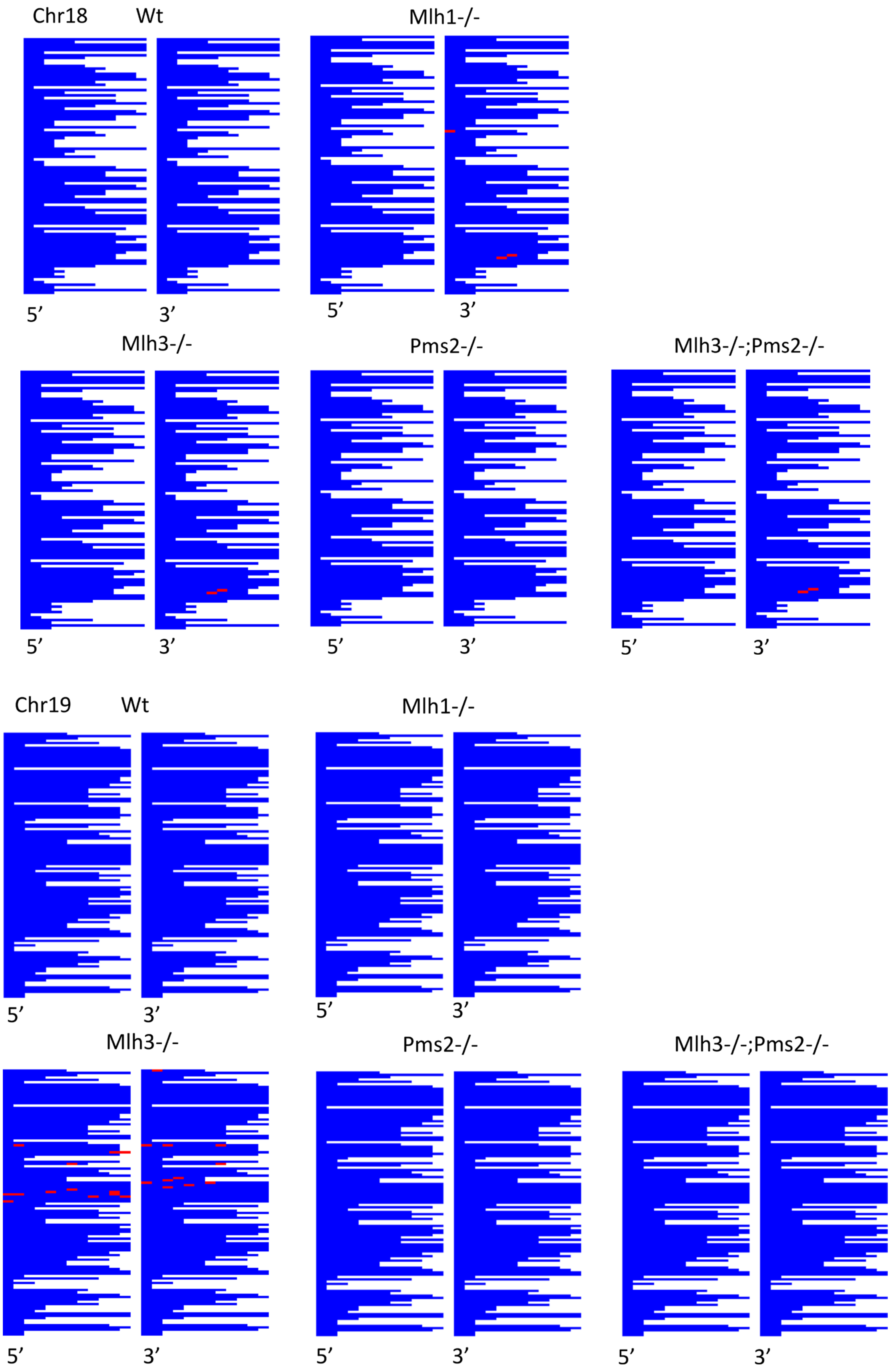

Supplementary Figure 3: Depiction of 7,863 mouse DR pairs with the following parameters: 5' and 3' DR motifs each > 480bp in the same orientation with 1-12 mismatched pairs (97%-99.9% identity) and intervening sequences ranging from 500bp to 50kb across the mouse genome.

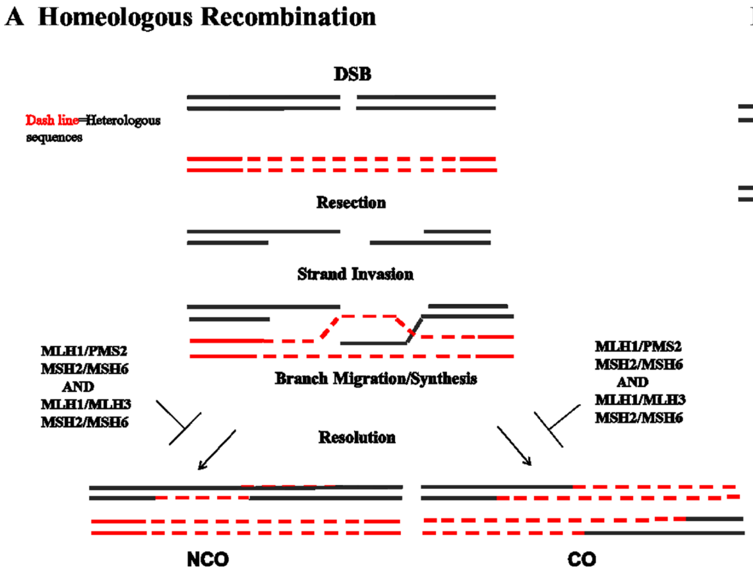

Supplementary Figure 4: Overview of MMR suppression of homeologous recombination. (**A**) MMR suppression of homeologous gene conversion. Sister chromatid exchange initiates homologous recombination DSB repair. The early steps of the reaction consist of single-strand resection and strand invasion, and are reviewed in detail elsewhere16. Both MLH1/PMS2/MSH2/MSH6 and MLH1/MLH3/MSH2/MSH6 MMR complexes recognize mismatched heterologous basepairs in double stranded DNA. Consequently, MMR suppresses both non-crossover (NCO) and crossover (CO) mediated resolution of homeologous DSB repair. (**B**) MMR suppression of homeologous single-strand annealing (SSA) DSB repair. DSBR occurs between heterologous direct repeats (red and blue boxes), then resection followed by annealing of the direct repeats results in SSA.
